# 736. Risk Factors and Outcomes Associated with Daptomycin-nonsusceptible Enterococcus Bloodstream Infections

**DOI:** 10.1093/ofid/ofad500.797

**Published:** 2023-11-27

**Authors:** Andrew J Failla, Geehan Suleyman

**Affiliations:** Henry Ford Hospital, Detroit, Michigan; Henry Ford Health, Detroit, Michigan

## Abstract

**Background:**

Enterococcus spp. are a common cause of nosocomial bloodstream infections (BSIs) with increasing resistance to currently available antibiotics and high mortality. Although the emergence of daptomycin-nonsusceptible Enterococcus (DNSE) has been reported, risk factors and outcomes associated with acquisition of daptomycin (DAP) resistance are not well characterized.

**Methods:**

Retrospective cohort study of patients with enterococcal BSIs at Henry Ford Health in Southeast Michigan from 2014 to 2022. Cases included patients with DNSE; patients with persistent daptomycin (DAP)-susceptible Enterococcus (DSE) bacteremia ( > 2 days of positive blood cultures) were used as controls. Outcomes included 30-day readmission and mortality.

**Results:**

24 cases and 24 controls were included; median age was 67 years (IQR 59-72), 28 (58.3%) were male and 26 (54%) white. All patients were exposed to antibiotics within 90 days with no difference in DAP use between the two groups (8% vs 21%, p=0.220). The majority (90%) had prior hospitalization within the year, 44% were immune suppressed and 42% had end-stage renal disease. Hepatitis C virus (HCV) (17% vs 0, 0.037) and prior VRE (42% vs 21%, p < 0.001) were more prevalent among cases. Indwelling urinary catheter (IUC) use was more common in cases (83% vs 54%, p=0.029), but there was no difference in central venous catheter use (75% vs 58%, p=0.221). Most common source of infection was intra-abdominal in both groups (54% vs 75%, p=0.188). Although not statistically significant, need for intensive care unit admission was higher (75% vs 50%, p=0.074) and length of stay was longer (54 vs 32 days, p=0.063) in cases compared to controls; treatment duration was significantly longer in controls (23 vs 16 days, p=0.04). There was no significant difference in readmission (12.5% vs 25%, p=0.267) or mortality (58% vs 46%, p=0.386) between the two groups.

Table 1

**Figure:**
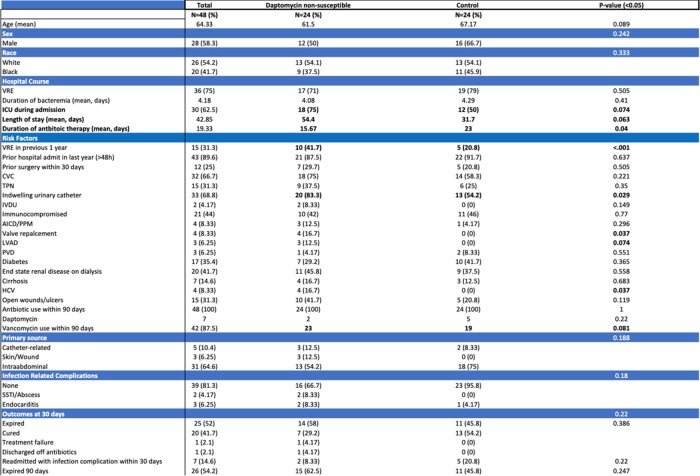

DNSE patient demographics, risk factors and outcomes

**Conclusion:**

DNSE is an emerging pathogen associated with HCV, prior VRE and IUC use in our cohort; however, prior daptomycin exposure was not a significant risk factor for DNSE. Despite the high mortality, there was no difference in outcome between the two groups. Mechanism of DNSE in patients without prior DAP exposure should be explored to prevent potential spread of resistance.

**Disclosures:**

**All Authors**: No reported disclosures

